# The first reported case of Aerococcus bacteremia in a patient with HIV infection.

**DOI:** 10.3201/eid0506.990622

**Published:** 1999

**Authors:** J H Razeq, G M Thomas, D Alexander


6. Giacomini T, Lusina D, Foubard S, Baledent F, Guibert F, Le Pennec MP. Dangers of hematological automated analysis for malaria diagnosis. Bull Soc Pathol Exot 1991;84:330-3.
7. de Grooth BG, Terstappen LW, Puppels GJ, Greve J. Light-scattering polarization measurements as a new parameter in flow cytometry. Cytometry 1987;8:539-44.
8. Mendelow BV, Lyons C, Nhlangothi P, Tana M, Munster M, Wypkema E, et al. Automated malaria detection by depolarization of laser light. Br J Haematol 1999;104:499-503.
9. Day NPJ, Thi Diep P, Thi Ly P, Xuan Singh D, Phu Loc P, Van Chuong L, et al. Clearance kinetics of parasite and pigment-containing leukocytes in severe malaria. Blood 1996;88:4694-700.
10. Lawrence C. Laveran remembered: malaria haemazoin in leucocytes. Lancet 1999;353:1852.
11. Phu NH, Day N, Thi Diep P, Ferguson DJP, White NJ. Intraleucocytic malaria pigment and prognosis in severe malaria. Trans R Soc Trop Med Hyg 1995;89:200-4.

### The First Reported Case of *Aerococcus* Bacteremia in a Patient with HIV Infection

**To the Editor:** We report the first case of *Aerococcus viridans* bacteremia in a patient with HIV infection. Two species in the genus *Aerococcus* have been implicated as rare pathogens in humans. *A. urinae* causes urinary tract infections; the other species, *A. viridans*, a gram-positive coccus considered a contaminant in cultures, has been associated with human infections that included bacteremia (1,2), septic arthritis (3), and infectious endocarditis (4,5). Widely distributed in the environment, the organism has been recovered from dust, vegetables, and crustaceans (6) and was isolated from different areas in a hospital (recovery room, intensive care unit, delivery room, treatment room, premature nursery) and from numerous objects (7).

We describe the first case of *A. viridans* bacteremia in a patient with HIV. A 34-year-old man without notable medical history sought medical attention after several weeks of epigastric midabdominal pain associated with a 15-lb weight loss; the pain did not respond to antacid medications. The patient said that he did not have fever, chills, night sweats, or history of transfusions and did not use alcohol, tobacco, or drugs. He had engaged in homosexual activity 2 to 3 years earlier.

Physical examination showed moderate cachexia and low-grade fever (38.8°C) associated with tachycardia, but the heart and lung examination was otherwise normal. The abdomen was soft, flat, and tender to palpation in the midabdominal epigastric area, without hepatosplenomegaly, guarding, or rebound tenderness. No other abnormalities were identified. The patient was admitted to the hospital, and the initial set of routine blood cultures (Bactec 9240 instrument, Becton Dickinson, Sparks, MD) showed no growth. On hospital day 2, he began to have severe rigors, along with persistent fever. A second set of blood cultures drawn at that time grew paired gram-positive cocci in less than 24 hours. The patient was empirically started on penicillin G, and cefotaxime was added shortly thereafter because of the possibility of intermediately resistant pneumococcus. The rigors responded to antibiotic treatment, and a third set of blood cultures showed no growth. The negative blood cultures before and after appropriate antimicrobial therapy and the short time to detection (which suggests a large initial inoculum) led us to believe that the organism in this case was a true pathogen and not a contaminant.

The patient's work-up included a normal abdominal computer tomography; abdominal ultrasound showed nonobstructing cholelithiasis. Laboratory tests demonstrated anemia of chronic disease diagnosed by a hematocrit of 25% associated with a low reticulocyte production index, high serum ferritin, and an elevated erythrocyte sedimentation rate (91 mm/hr), with polyclonal hypergammaglobulinemia and hypoalbuminemia on serum protein electrophoresis. Stool samples were negative for occult blood, and serologic tests showed no *Helicobacter pylori* antibodies. The patient's total lymphocyte count was 300 cells/µl, HIV serologic testing by enzyme-linked immunosorbent assay and Western blot was positive, and flow cytometry revealed an absolute CD4+ T-lymphocyte count of 19 cells/µl, with an HIV-1 retroviral titer of 280,000 by polymerase chain reaction. Gallium scanning was negative for *Pneumocystis carinii* pneumonia and gastrointestinal lymphoma. A follow-up endoscopy showed esophageal ulcers, with disruption of the mucosal barrier. Blood cultures were negative for cytomegalovirus or mycobacteria, but the aerobic isolate initially reported as paired gram-positive cocci was later identified as *A. viridans.*

The identification of *A. viridans* was made on the basis of the following characteristics: catalase negativity, α-hemolytic gram-positive cocci forming pairs and tetrads (not chains) in broth culture; growth in the presence of 40% bile and 6.5% NaCl and ability to hydrolyze esculin; pyrrolidony l-aminopeptidase positivity, leucine-aminopeptidase negativity; and production of acid from trehalose, sucrose, maltose, and lactose but not from sorbitol.

Susceptibility testing by the agar dilution method showed that the isolate was susceptible to penicillin-G (MIC = 0.12 µg/ml) and vancomycin (MIC = 0.25 µg/ml). On the basis of this case and previous reports (1,2), we believe that *A. viridans* is a potential pathogen that can cause serious infections in immunocompromised patients. The presumed route of infection in this patient was esophageal ulcers. Clinical microbiologists should pay close attention to α-hemolytic, catalase-negative streptococci recovered from sterile body sites that form tetrads rather than chains on Gram stain.

**Jafar H. Razeq,* Gloria M. Thomas,* and Daniel Alexander†**

*State of Maryland Department of Health and Mental Hygiene Laboratories Administration, Baltimore, Maryland, USA; †Franklin Square Hospital, Baltimore, Maryland, USA

### References

1. Swanson H, Cutts E, Lepow M. Penicillin-resistant *Aerococcus viridans* bacteremia in a child receiving prophylaxis for sickle-cell disease. Clin Infect Dis 1996;22:387-8.
2. Kern W, Vanek E. *Aerococcus* bacteremia associated with granulocytopenia. European Journal of Clinical Microbiology 1987;6:670-3.
3. Taylor PW, Trueblood MC. Septic arthritis due to *Aerococcus viridans*. J Rheumatol 1985;5:1004-5.
4. Untereker WJ, Hanna BA. Endocarditis and osteomyelitis caused by *Aerococcus viridans*. Mt Sinai J Med 1976;43:248-52.
5. Janosek J, Eckert J, Hudac A. *Aerococcus viridans* as a causative agent of infectious endocarditis. Journal of Hygiene, Epidemiology, Microbiology and Immunology 1980;1:92-6.
6. Pien FD, Wilson WR, Kunz K, Washington JA II. *Aerococcus viridans* endocarditis. Mayo Clin Proc 1984;59:47-8.
7. Kerbaugh MA, Evans JB. *Aerococcus viridans* in the hospital environment. Applied Microbiology 1968;16:519-23.

### Proficiency in Detecting Vancomycin Resistance in Enterococci among Clinical Laboratories in Santiago, Chile

**To the Editor:** Vancomycin-resistant enterococci (VRE) can be difficult to detect because of limitations in the susceptibility testing methods commonly used in clinical laboratories. Although VRE have not been reported in Chile, clinical isolates have been reported in Argentina (1) and Brazil (2). It is important to detect vancomycin resistance as early as possible, so infection control preventive measures can be instituted when they have their greatest impact. The microbiology laboratory is the first line of defense against VRE, as it plays a critical role in its recognition. In Chile, most laboratories follow the National Committee for Clinical Laboratory Standards recommendations for antimicrobial susceptibility testing and use disk-diffusion methods (3); however, these methods have limitations in detecting low levels of resistance to vancomycin in enterococci.

We evaluated the ability of referral microbiology laboratories in Chile to detect vancomycin resistance in five *Enteroccocus* spp. isolates with different susceptibility patterns for vancomycin, penicillin, and ampicillin. Of six referral laboratories that agreed to participate, four used the disk-diffusion method to evaluate antimicrobial susceptibility. Two used an agar dilution minimum inhibitory concentration (MIC) method, one as the only susceptibility testing method and the other in addition to disk diffusion. The participants correctly evaluated vancomycin susceptibility in 17 (57%) of 30 isolates.

The accuracy of detecting vancomycin resistance varied according to the level of resistance. Isolate 1, which had a high level of resistance (Van A phenotype, MIC 256 µg/ml), was evaluated correctly in 5 (83%) of 6 laboratories. Isolate 2, with a lower level of resistance (Van B, MIC 64 µg/ml), was evaluated correctly in 4 (67%) of 6 laboratories. Isolates 3 and 4, both with intermediate resistance (Van B, MIC 16-32 µg/ml, and Van C, MIC 8 µg/ml, respectively), were evaluated correctly by one laboratory each. Isolate 5 (vancomycin susceptible) was evaluated correctly by all laboratories. Susceptibility to penicillin and ampicillin was correctly identified in 53 (96.4%) of 55 isolates. Although laboratories

